# Distention of the Immature Left Ventricle Triggers Development of Endocardial Fibroelastosis: An Animal Model of Endocardial Fibroelastosis Introducing Morphopathological Features of Evolving Fetal Hypoplastic Left Heart Syndrome

**DOI:** 10.1155/2015/462469

**Published:** 2015-05-03

**Authors:** Shogo Shimada, Christian Robles, Ben M. W. Illigens, Alejandra M. Casar Berazaluce, Pedro J. del Nido, Ingeborg Friehs

**Affiliations:** Department of Cardiac Surgery, Boston Children's Hospital, Harvard Medical School, 300 Longwood Avenue, Boston, MA 02115, USA

## Abstract

*Background.* Endocardial fibroelastosis (EFE), characterized by a diffuse endocardial thickening through collagen and elastin fibers, develops in the human fetal heart restricting growth of the left ventricle (LV). Recent advances in fetal imaging indicate that EFE development is directly associated with a distended, poorly contractile LV in evolving hypoplastic left heart syndrome (HLHS). In this study, we developed an animal model of EFE by introducing this human fetal LV morphopathology to an immature rat heart. *Methods and Results.* A neonatal donor heart, in which aortic regurgitation (AR) was created, was heterotopically transplanted into a recipient adult rat. AR successfully induced the LV morphology of evolving HLHS in the transplanted donor hearts, which resulted in the development of significant EFE covering the entire LV cavity within two weeks postoperatively. In contrast, posttransplants with a competent aortic valve displayed unloaded LVs with a trace of EFE. *Conclusions.* We could show that distention of the immature LV in combination with stagnant flow triggers EFE development in this animal model. This model would serve as a robust tool to develop therapeutic strategies to treat EFE while providing insight into its pathogenesis.

## 1. Introduction

Endocardial fibroelastosis (EFE), characterized by a diffuse endocardial thickening through collagen and elastin fibers, predominantly develops in immature left atrium (LA) and left ventricle (LV) [1, 2]. EFE has been described in association with a wide variety of diseases, such as viral myocarditis [3], lysosomal storage diseases [4], idiopathic or genetic dilated cardiomyopathies [5, 6], immunologic diseases [7, 8], and structural cardiac malformations [9, 10], such as hypoplastic left heart syndrome (HLHS). Although it has been speculated that an early hemodynamic insult on the immature LV plays a key role in the development of EFE, its precise pathogenesis has not been elucidated [11, 12].

Recent advances in fetal imaging have demonstrated that in a subset of HLHS patients progression of fetal aortic stenosis occurs relatively late in gestation [13]. Fetal aortic balloon valvuloplasty (FAV) has been successfully performed to avert this progression in a selected patient population [14]. The development of EFE has been well described in the progression of this disease and has been shown to contribute to LV growth retardation and immediate, and more likely, long-term diastolic dysfunction [15, 16]. Disease progression is characterized by dramatic morphological changes of the affected LV. Initially, the LV appears normal in size with decreased contractility; then, it develops dilation with hyperechogenic endocardium, indicative of EFE. Finally, later in gestation, it progresses into a hypocontractile state with LV hypoplasia that meets the diagnostic criteria for HLHS [17]. These clinical observations of the LV suggest that distention is a key factor in the development of EFE. In addition, it has been recognized that EFE is more likely to develop in a borderline developed LV rather than a diminutive LV, the latter being protected from distention since it has neither inlet nor outlet [16, 18, 19]. Furthermore, most of LVs developing EFE from other etiologies are distended at the time of EFE manifestation [1, 2, 9, 20]. Collectively, these clinical observations implicate that distention of the immature LV may significantly add to the severity of EFE development, potentially on the basis of unknown intrinsic genetic or immunologic predispositions.

Based on our hypothesis that immaturity and stagnation of intracavitary flow would play a key role in the development of EFE, we reported our results on EFE development in a heterotopically transplanted neonatal rat heart model, where the LV had no intracavitary flow (i.e., preload) [21]. This animal model, however, showed variable degrees of EFE formation which we could only assess at postmortem analysis since echocardiographic evaluation was limited due to restricted view of the intra-abdominally located donor heart. In addition, postmortem analysis indicated a direct correlation between the degree of EFE and ventricular distention which was most likely a result of intraoperative technical difficulties resulting in the distortion of the aortic root. These combined observations of clinical and experimental data indicate that additional factors contribute to the pathophysiological mechanism of EFE formation. Thus, we refined our hypothesis, combining immaturity and stagnation of flow within the LV cavity with intentional LV distention and modification of the anatomical location of the heterotopically transplanted heart for echocardiographic monitoring. In order to test this hypothesis, we modified the previously described heterotopically transplanted immature heart model by introduction of acute LV distention through creation of severe aortic regurgitation without compromising coronary perfusion and femoral location of the donor graft.

## 2. Materials and Methods

All animal procedures in this study were conducted in accordance with the Principles of Laboratory Animal Care formulated by the National Society for Medical Research and the Guide for the Care and Use of Laboratory Animals prepared by the Institute of Laboratory Animal Resources and published by the National Institutes of Health (NIH Publication number 86-23, revised 1996). The animal protocols were reviewed and approved by the Institutional Animal Care and Use Committee at Boston Children's Hospital.

### 2.1. Heterotopic Femoral Neonatal Rat Heart Transplantation

Heterotopic femoral heart transplantations were performed between syngeneic Lewis rats (Charles River Laboratories International, Wilmington, MA) using the technique previously described with a few modifications [22]. The procedures were performed under a surgical microscope (Endure Medical, Georgia) with 6x to 40x magnification. Donors with AR creation served as a distended LV model (*n* = 10) and those with an intact aortic valve served as unloaded LV model (*n* = 14).

#### 2.1.1. Recipient Preparation

Recipient rats (male, 150 to 200 g) were anesthetized via intraperitoneal injections of Ketamine (40 mg/kg) and Xylazine (10 mg/kg). Heparin (300 IU/kg) was also administered intraperitoneally. Anesthesia was maintained by isoflurane inhalation (1 to 2%) through an endotracheal tube under mechanical ventilation (Inspira Advanced Safety Ventilator, Harvard Apparatus, Holliston, MA). A skin incision was made along the inguinal crease to expose the femoral artery and vein.

#### 2.1.2. Donor Preparation and Creation of Aortic Regurgitation

Neonatal rats (postnatal day 2 to 4, 10 ± 2 g) served as a donor. Anesthesia was induced in the same way as in the recipients. The chest was opened with a V-shaped incision to free the entire anterior rib cage for wide exposure. High potassium Krebs-Henseleit solution was administered via the inferior vena cava to obtain optimal cardiac preservation and eliminate blood. The distal ascending aorta and pulmonary trunk were cut and divided as proximally as possible to facilitate subsequent anastomoses. All other accessory vessels were ligated by 7-0 nylon sutures and cut off. The aortic valve was either damaged by inserting an ultrathin guide-wire (Roadrunner Extra-Support Wire Guide, diameter: 0.014 inch, COOK MEDICAL, Bloomington, IN) to create aortic regurgitation or left intact. The harvested heart was stored in cardioplegic solution at 4°C.

#### 2.1.3. End-to-End Anastomoses

**Figure 1:**
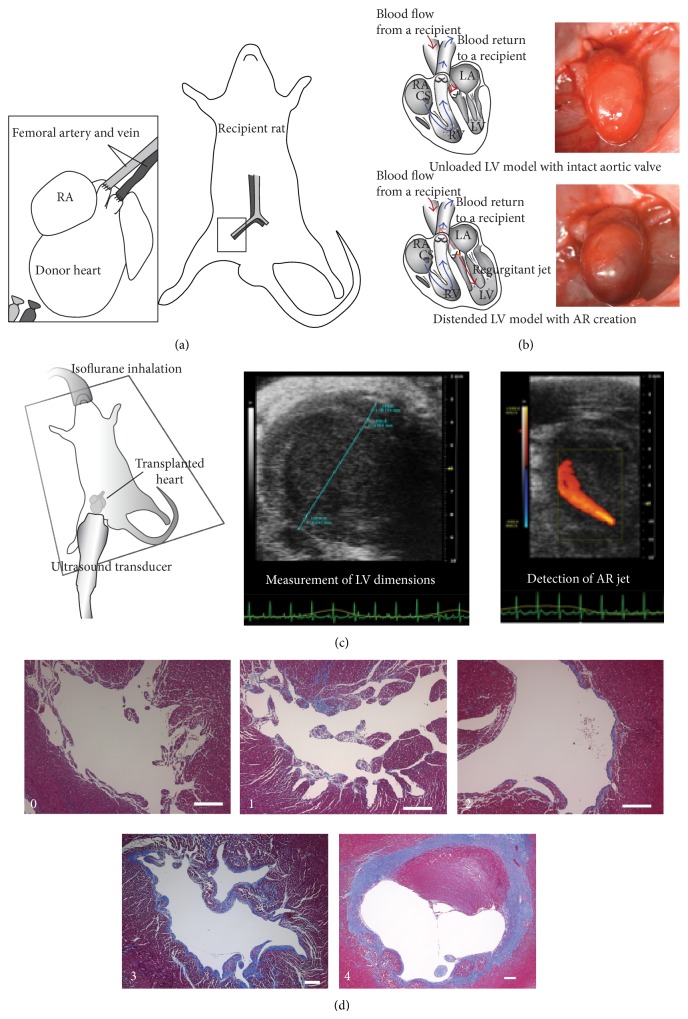
(a) Heterotopic femoral heart transplantation. (b) Schemes of blood circulation of transplanted hearts and representative photographs of the posttransplants. Reduced flow through the LV in the unloaded LV model with intact aortic valve (upper row) versus increased pressure and volume load in the distended LV model with AR creation (lower row). (c) Postoperative trans-femoral echocardiography. Representative images from a distended LV with AR. (d) A grading system for the amount of EFE. EFE scores (Grade 0, no EFE; Grade 1, islets of EFE; Grade 2, thin EFE tissue covering a half circumference of the endocardium; Grade 3, thin EFE tissue (less than 100 *μ*m) covering a full circumference of the endocardium; Grade 4, thick EFE tissue (more than 100 *μ*m) covering a full circumference of the endocardium). White scale bar, 200 *μ*m.

Microvascular clamps were placed on the recipient proximal femoral artery and vein separately. The distal femoral artery and vein were tied off before branching superficial caudal epigastric artery and vein. The harvested donor heart was then transferred into the recipient groin for transplantation. The donor's ascending aorta and pulmonary trunk were anastomosed to the recipient's femoral artery and vein, respectively, in an end-to-end manner. The arterial anastomosis was made by 8 to 10 interrupted stitches with 10-0 and 11-0 nylon sutures. The venous anastomosis was made by continuous running suture with an 11-0 nylon suture. After completion of the anastomoses, the microvascular clamps were removed. The transplanted hearts resumed beating within a minute and showed variable distention according to the degree of aortic regurgitation. The incision was closed following hemostasis. The recipient rat was then given analgesics (Buprenorphine: 0.1 mg/kg S.C., Meloxicam: 1 mg/kg S.C.) and extubated. The rat was allowed to recover and usually had no difficulty in ambulation. No limitation on feeding was imposed perioperatively and no antibiotics were given (Figures 1(a) and 1(b)).

### 2.2. Heterotopic Abdominal Rat Heart Transplantation

Conventional heterotopic abdominal heart transplantations using a 2-week-old donor with AR creation were performed as previously described (*n* = 3) [23, 24]. Briefly, the donor heart harvest and AR creation was carried out in the same way as the femoral transplantation. The donor's great vessels were anastomosed to a recipient's infrarenal abdominal aorta and inferior vena cava, respectively, in an end-to-side manner. Postoperative care and time course of the experiments were the same as the femoral transplantation.

### 2.3. Postoperative Trans-Femoral Echocardiography

The transplanted hearts underwent echocardiographic evaluation for LV dimensions, contractility, and aortic valvular function one week postoperatively. The recipient rat was anesthetized by isoflurane inhalation (1 to 2%) delivered via a nose cone and positioned supine on a heated platform for echocardiography (Vevo 2100, FUJIFILM VisualSonics, Toronto, Canada). A long axis view of the transplanted heart was visualized through an apical approach by a 40 MHz transducer (MS550D, FUJIFILM VisualSonics, Toronto, Canada). Data were acquired via this apical long axis view (Figure 1(c)).

### 2.4. Histological Evaluation of the Transplanted Hearts

The recipient rats were euthanized two weeks postoperatively. The transplant hearts were explanted and fixed in 4% paraformaldehyde for 24 hours, embedded in paraffin, and sectioned to obtain either short or long axis view of the LV. Hematoxylin and Eosin staining, Masson's Trichrome staining, and Elastica van Gieson staining were performed on those sections using standard protocols to determine EFE. The degree of EFE was graded semiquantitatively on a scale from 0 to 4 (Grade 0, no EFE; Grade 1, islets of EFE; Grade 2, thin EFE tissue covering half of the circumference of the LV endocardium; Grade 3, thin EFE tissue (less than 100 *μ*m) covering the full circumference of the LV endocardium; Grade 4, thick EFE tissue (more than 100 *μ*m) covering the full circumference of the LV endocardium; Figure 1(d)). Images were acquired on a microscope (Axio Observer. Z1, Carl Zeiss Microscopy LLC, Peabody, MA).

### 2.5. Statistical Analysis

LV parameters of the posttransplants from echocardiographic measurements were assessed for group differences using an unpaired *t*-test. Comparisons of EFE scores between groups were made using nonparametric (Mann-Whitney) tests conducted with JMP (8.0.1, SAS Institute, Japan). Data are expressed as means ± SEMs. *P* < 0.05 was considered statistically significant.

## 3. Results

### 3.1. AR Successfully Induced the LV Morphology of Evolving HLHS on the Transplanted Donor Hearts

**Figure 2:**
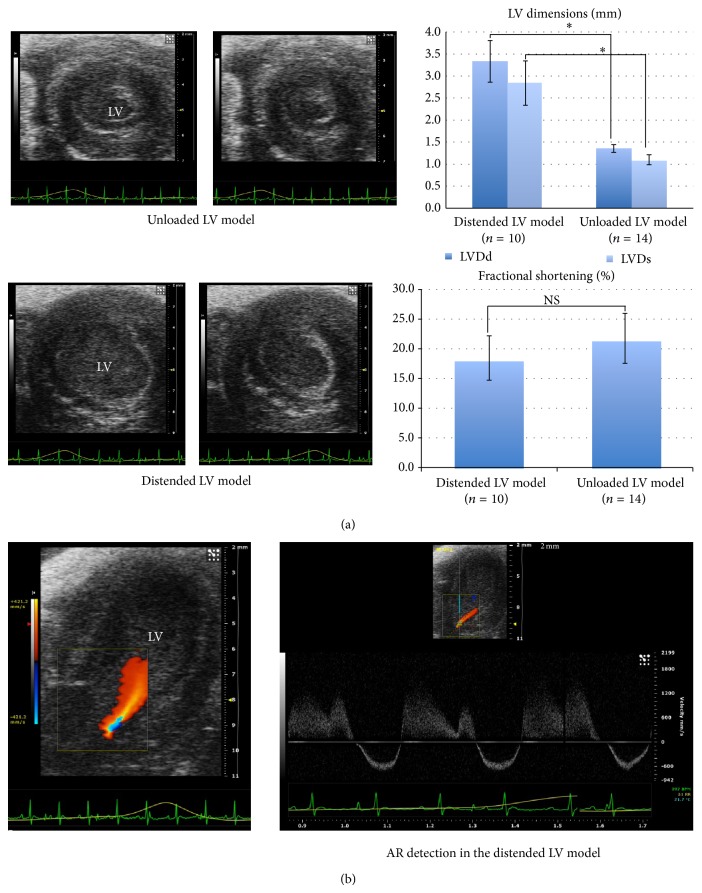
Postoperative echocardiography. (a) Short axis views of LVs in diastole and systole. The distended LV model developed significantly larger LVs than those in the unloaded LV model (^*^ *P* < 0.01, NS: not significant). (b) Apical long axis view of a LV in the distended LV model. An AR jet was detected in a 2D echocardiography with color Doppler.

We conducted heterotopic femoral neonatal rat heart transplantations to test if AR creation could induce acute LV distention on the immature neonatal donor rat heart. The transplanted hearts with AR creation (*n* = 10) were compared to those with an intact aortic valve (*n* = 14) via postoperative trans-femoral echocardiography. In the distended LV model, the LVs were markedly dilated by significant AR, whereas those in the unloaded LV model became contracted with reduced preload (LVDd (mm), 3.33 ± 0.47 versus 1.35 ± 0.09, *P* < 0.01; LVDs (mm), 2.84 ± 0.50 versus 1.10 ± 0.12, *P* < 0.01) (Figure 2(a)). Both groups showed decreased LV contractility without any significant difference (FS (%), 18.45 ± 3.73 versus 21.77 ± 4.22, N.S.). These echo findings indicate that AR introduction could instantaneously alter the morphology of the transplanted hearts due to significant pressure and volume overload.

### 3.2. EFE Developed in the Distended Immature LVs

**Figure 3:**
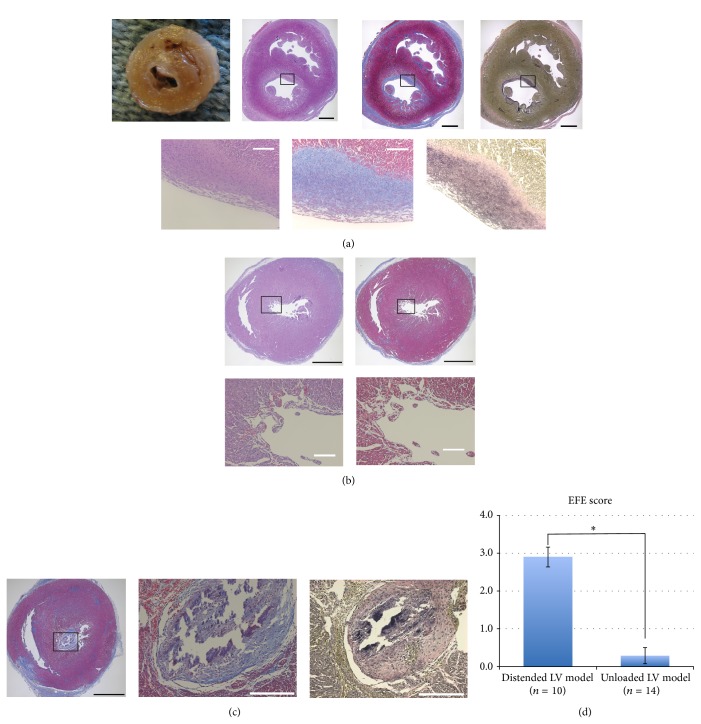
(a) A representative photograph and slides of the distended LV model. Each shows short axis views of the ventricles. The endocardium is covered by macroscopically discernible pearly white thick layers of fibroelastosis. Masson's Trichrome- and Elastica van Gieson-stained slides depict thick fibrous layers with an abundant collagen deposition (stained in blue) and stratified elastin fibers (black wavy lines) on the endocardial surface. (b) Representative Hematoxylin and Eosin- and Masson's Trichrome-stained slides in the unloaded LV model. The LV appears contracted with a thickened myocardial layer. No apparent fibrous tissue develops on the endocardium. (c) A case with a mural thrombus in the unloaded LV model. Masson's Trichrome-stained slides depict a large mural thrombus occupying the LV cavity. The mural thrombus has an abundant collagen deposition but no elastin fibers in an Elastica van Gieson-stained slide. Black scale bar, 1 mm; white scale bar, 200 *μ*m. (d) Comparison of EFE scores between the distended LV model and the unloaded LV model (^*^ *P* < 0.01).

Histological evaluation was performed to determine if these distended hearts developed significant EFE. In the distended LV model with AR, the explanted hearts were larger in size and all 10 cases developed significant EFE. In the most severe EFE cases (EFE score 4), EFE was macroscopically discernible as a thick white layer on the endocardial surface. Microscopic observations confirmed a collagen-rich fibrous tissue with elastin fibers covering the endocardial surface, which specified EFE (Figure 3(a)). In contrast, the unloaded LVs appeared contracted, and EFE developed only in 2 cases. Instead, a mural thrombus was organized in 3 out of 14 unloaded LVs and distinguished from EFE by the absence of elastin fibers (Figures 3(b) and 3(c)). EFE scores in the distended LV model were significantly higher than those in the unloaded LV model (2.90 ± 0.26 versus 0.29 ± 0.21, *P* < 0.01) (Figure 3(d)).

**Figure 4:**
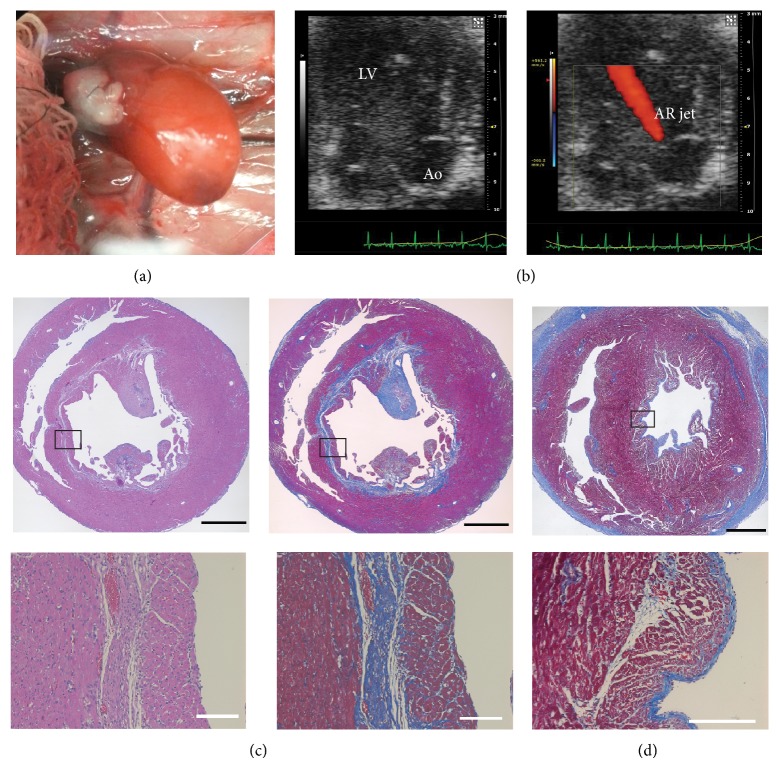
In comparison, the distended LV model using a 2-week-old donor. (a) A photograph of a transplanted donor heart in situ. (b) Representative echo images showing the apical long axis view of the LV. An AR jet was detected in a 2D echocardiography with color Doppler. (Ao: aorta). (c) Representative Hematoxylin and Eosin- and Masson's Trichrome-stained slides sectioned along the short axis. Collagen-rich fibrous layers (stained in blue) are seen in the subendocardial layer of a 2-week-old donor heart. (d) A Masson's Trichrome-stained slide from a neonatal donor heart for comparison. Fibrous layers are located on the endocardial surface of the LV. Black scale bar, 1 mm; white scale bar, 200 *μ*m.

EFE develops almost exclusively in the early stage of life. We, therefore, performed the distended LV model using a relatively mature donor (2-week-old, *n* = 3) (Figures 4(a) and 4(b)). In this model, none of the transplanted hearts developed EFE, and fibrosis was mainly distributed in the subendocardial layer instead of the endocardial surface (Figure 4(c)).

### 3.3. EFE Increased in Proportion to the Severity of LV Distention

**Figure 5:**
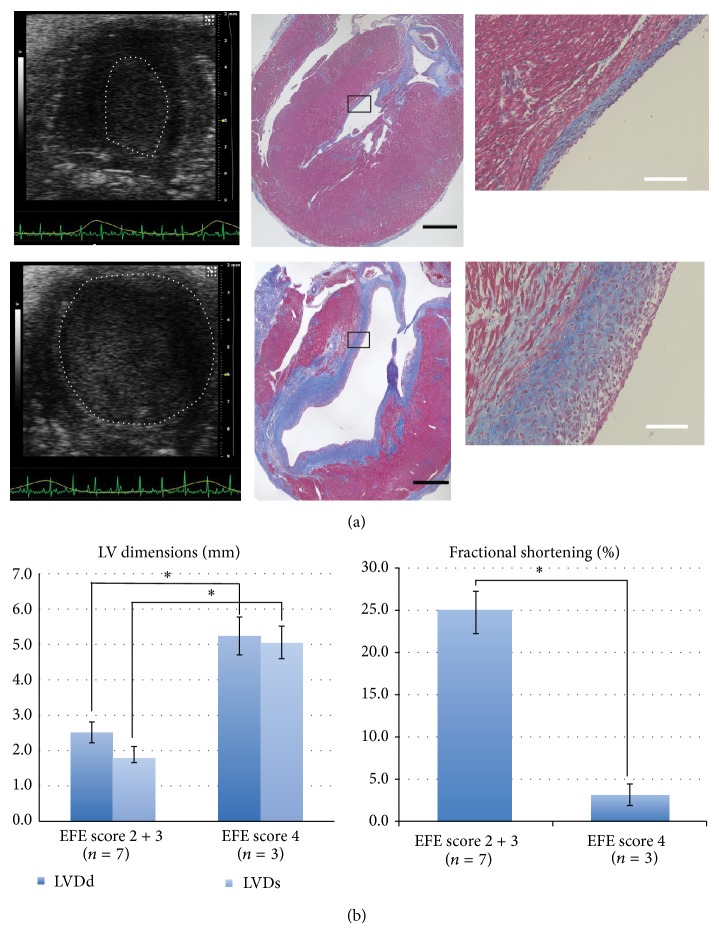
Relationship between LV distention and EFE amount. (a) Representative echo images and Masson's Trichrome-stained slides sectioned along the long axis. Mild LV distention created mild EFE (Grade 2) (upper row), whereas severe LV distention created severe EFE (Grade 4) (lower row). White dots in the echo images delineate the contour of the LV endocardium. Black scale bar, 1 mm; white scale bar, 200 *μ*m. (b) Comparison of the LV dimensions and fractional shortenings between Grade 2, 3 EFE cases and Grade 4 EFE cases (^*^ *P* < 0.01).

Although all the distended LVs developed significant EFE, its degree varied. To further clarify the relationship between distention and EFE development, we compared echo data between the most severe EFE cases (Grade 4, *n* = 3) and the others (Grade 2 and 3, *n* = 7) from the distended LV model. Echo analyses revealed that the most severe degree of EFE (Grade 4) developed in the most dilated (LVDd (mm), 5.24 ± 0.54 versus 2.51 ± 0.29, *P* < 0.01; LVDs (mm), 2.84 ± 0.50 versus 1.10 ± 0.12, *P* < 0.01) and poorly contractile LVs (FS (%); 3.15 ± 1.28 versus 25.0 ± 2.77, *P* < 0.01) (Figure 5). The severity of distention was directly associated with the amount of EFE.

## 4. Discussion

In this study, we successfully created an animal model of EFE by introducing clinical features of evolving human fetal HLHS which included a combination of immaturity, stagnation of flow, and distention of the LV through volume/pressure overload. Our results indicate that the amount of EFE directly correlates with the severity of LV distention when added to immaturity and stagnation of intracavitary flow. These findings shed new light on the underlying mechanisms of EFE development, which are in line with clinical observations of fetal patients with aortic stenosis and impending HLHS development.

Although multiple animal models have produced LV hypertrophy by introducing left-sided obstruction, none have been successful in creating EFE [25, 26]. Major limitations using fetal experimental animals are difficulties in obtaining survival cases when applying major hemodynamic changes acutely. Creating aortic stenosis in an early gestational fetus gradually increases LV end-diastolic pressure but fails to create abrupt augmentation of LV pressure/volume load, which we observed in a subgroup of fetuses with aortic stenosis and evolving HLHS. Thus, as a consequence, no EFE developed in these animal models [25].

Fully vascularized cardiac transplantation in small animals has been established and reliably used to address a wide variety of issues. Multiple modifications have been attempted to facilitate construction of the anastomoses, which has been recognized as a technical challenge and requires a substantial learning curve to achieve acceptable reproducibility [27]. Using a neonatal rat as a donor would be even more challenging given the small size and fragility. To overcome this potential vulnerability of the present model, postoperative monitoring was imperative. Therefore, we newly developed a heterotopic femoral heart transplantation model, in which a transplanted heart was vascularized by the recipient's femoral vessels and placed superficially in a groin pocket. Unlike the conventional abdominal transplantation model, this model made the posttransplant heart palpable and visible via postoperative trans-femoral echocardiography.

In the present study, the unloaded LV model did not produce significant EFE as indicated by our scoring data. The discrepancy toward our previously published results may be attributable to subtle technical differences, such as the way of placing a stitch, aligning the target vessels, positioning the posttransplant with possible distortion of the aortic root, and obtaining optimal hemostasis, given the nature of this technically demanding and thus fairly surgeon-dependent procedure. Technical differences resulted in altering degrees of compromised LV function, which potentially influenced EFE formation. We addressed this issue by refining our animal model through incorporation of LV distention, which provided reproducible degrees of LV dysfunction. Alternatively, it can be attributed to diagnostic challenges with EFE. A mural thrombus or other nonspecific endocardial fibrosis could mimic EFE. In particular islet-type patchy fibrosis on the endocardium is difficult to determine due to the phenotypical ambiguity and indiscernible elastin fibers.

To fulfill distention of the LV, we introduced AR through direct damage of the aortic valve. AR mimics the clinical situation of a distended LV in some impending HLHS cases but does not occur as a prominent pathophysiologic feature in human fetuses. Thus, there is the potential that the direction and velocity of blood flow following AR through the LV or shear stress applied onto the endocardium could be completely different. The AR jet might, thereby, directly injure the endocardium and induce EFE formation regardless of LV distention. Since EFE in this study is distributed homogeneously over the full circumference of the endocardium, our results indicate that the AR jet does not directly injure the endocardium. Therefore, direct injury from an AR jet, which would have created spatially variable local lesions, is an unlikely contributor to EFE formation in this model. Direct localized endothelial injury could also have been caused by accidental trauma through the needle used for damaging the aortic valve leaflet. Initial attempts, in which we used a rigid thin needle to create AR, resulted in a localized fibrous scar lesion at the location of the injured endocardium (data not shown). This technical failure prompted us to use a flexible ultrathin guide-wire to create AR, which eliminated the risk of direct injury to the endocardium.

Difficulty in regulating the degree of AR generates different degrees of LV distention and results in variable amounts of EFE formation but at the same time provides a positive relationship between LV distention and the amount of EFE. Variable AR amounts also impose a challenge on a direct comparison between an immature donor group (P 2 to 4) and a relatively mature donor group (2-week-old) since the severity of LV distention, which is represented by the size and function of the LV, is not directly comparable. In addition, susceptibility to distention may vary. However, the fact that the distended LV model with a 2-week-old donor created prominent subendocardial fibrosis but no EFE (Figure 4(c)) indicates that not only distention but also immaturity plays a key role in this pathological process. To the best of our knowledge, none of the animal models of AR using adult subjects have described EFE as a histological change [28, 29].

It is still controversial whether EFE is a distinct pathologic entity or a secondary phenomenon caused by stressors on the heart, such as mechanical overload. In order to explore benefits and limitations of therapeutic interventions on the immature/fetal heart it is imperative to create a robust animal model of EFE to elucidate the underlying cause of EFE formation and to determine effective therapeutic agents targeting EFE. Intuitively, suppression of overgrowth of fibrotic layers on the endocardium, preferably combined with the mechanical relief of the distention such as FAV during the fetal stage, would provide a better chance to use the LV as systemic ventricle. Indeed, in experienced institutions, postnatal surgical resection of EFE has been performed in an attempt to restore and recruit the LV and improved outcome in selected cases has been reported [30, 31]. However, given the possibility that EFE is a protective response of the immature heart from mechanical overload, it might not be a useful therapeutic target during fetal development.

The current model shows that distention of the ventricle is one of the important mechanical factors which triggers the development of EFE in an age-dependent manner. These findings implicate that a relief of the underlying mechanical overload such as fetal aortic stenosis is of primary importance to mitigate EFE formation and exerts the greatest benefit if performed promptly when the mechanical overload appears. Since immediate relief of the mechanical overload to prevent EFE formation might not be feasible, alternative treatment strategies should potentially dissolve or reverse already present EFE.

## 5. Conclusions

We present an animal model where we could show that the distention of the immature LV triggers EFE formation by introducing morphopathological features of evolving human fetal HLHS. This model could serve as a robust tool to develop therapeutic strategies to treat EFE while providing insight into its pathogenesis.
